# Bacterial Antibiotic Resistance: The Most Critical Pathogens

**DOI:** 10.3390/pathogens10101310

**Published:** 2021-10-12

**Authors:** Giuseppe Mancuso, Angelina Midiri, Elisabetta Gerace, Carmelo Biondo

**Affiliations:** 1 Department of Human Pathology, University of Messina, 98125 Messina, Italy; mancusog@unime.it (G.M.); amidiri@unime.it (A.M.); 2 IRCCS Centro Neurolesi “Bonino-Pulejo”, 98100 Messina, Italy; geraceelisabetta74@gmail.com

**Keywords:** multidrug-resistant, carbapenem-resistant, ESBL, ESKAPE

## Abstract

Antibiotics have made it possible to treat bacterial infections such as meningitis and bacteraemia that, prior to their introduction, were untreatable and consequently fatal. Unfortunately, in recent decades overuse and misuse of antibiotics as well as social and economic factors have accelerated the spread of antibiotic-resistant bacteria, making drug treatment ineffective. Currently, at least 700,000 people worldwide die each year due to antimicrobial resistance (AMR). Without new and better treatments, the World Health Organization (WHO) predicts that this number could rise to 10 million by 2050, highlighting a health concern not of secondary importance. In February 2017, in light of increasing antibiotic resistance, the WHO published a list of pathogens that includes the pathogens designated by the acronym ESKAPE (*Enterococcus faecium, Staphylococcus aureus, Klebsiella pneumoniae, Acinetobacter baumannii, Pseudomonas aeruginosa,* and *Enterobacter species*) to which were given the highest “priority status” since they represent the great threat to humans. Understanding the resistance mechanisms of these bacteria is a key step in the development of new antimicrobial drugs to tackle drug-resistant bacteria. In this review, both the mode of action and the mechanisms of resistance of commonly used antimicrobials will be examined. It also discusses the current state of AMR in the most critical resistant bacteria as determined by the WHO’s global priority pathogens list.

## 1. Introduction

Although antimicrobial resistance (AMR) is a natural process, the public health emergency due to the uncontrolled spread of this phenomenon depends primarily on the overuse of antibiotics [1]. However, other factors are also primarily responsible for the increase in its prevalence [2]. These factors commonly referred to as "socioeconomic determinants" include poor community hygiene, safer food, poor infection control in hospitals and clinics, accumulation of antibiotics in the environment and their use in the animal and food industries [2]. Bacterial resistance to antibiotics was already known more than 50 years ago since, by the late 1950s, most isolates of *S. aureus* developed resistance to penicillin that in the past had normally been used to treat them [3]. Nevertheless, for a long time, antibiotic resistance was not a serious concern worldwide since, in the 1960s, new classes of drugs have been developed, such as vancomycin and methicillin, which suggested that the problem of resistance might be easily solved through the synthesis of new molecules [4]. Unfortunately, in the following decades, bacteria developed many different antibiotic resistance mechanisms that have protected them from the effects of these drugs and consequently antibiotic resistance has moved on [5]. In 2017, the World Health Organization (WHO) first published a list of 12 families of bacteria that pose the greatest threat to human health [6]. The WHO’s list categorizes bacteria into three categories of priority: critical, high and medium priority, according to the urgency of need to develop new antibiotics to combat these pathogens [7]. The pathogens included in the most critical group are multidrug resistant bacteria that pose threats to patients in hospitals and nursing homes as well as to patients whose conditions require medical devices such as ventilators and blood catheters [8,9]. Critical-priority bacteria comprise *Acinetobacter,* *Pseudomonas*, some Enterobacteriaceae such as: *K. pneumoniae*, *E. coli* and *Enterobacter spp*. [10]. These pathogens are resistant to multiple antibiotics and can cause severe and often fatal infectious diseases such as bloodstream infections and pneumonia [9]. The high priority category includes bacteria such as *Enterococcus faecium* and *Staphylococcus aureus* that are resistant to various antibiotics, such as vancomycin and fluoroquinolones. The medium priority category includes bacteria such as *Streptococcus pneumoniae* and *Shigella* that, although they may have some resistance, effective antibiotics are still available that can kill them [9,11]. 

In 2019, due to its impact on human health, the World Health Organization (WHO) included antimicrobial resistance (AMR) as one of the top ten threats to global health [12]. 

## 2. What Is It and What Are the Mechanisms by Which Antimicrobial Resistance Is Steadily Increasing?

**Figure 1:**
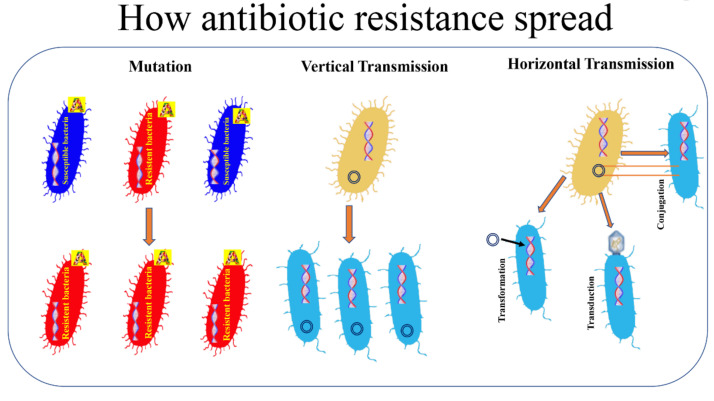
How antibiotic resistance spread. Bacterial resistance towards antibiotics can be natural, or acquired by vertical or horizontal transmission. A: antibiotic.

According to the World Health Organization, antimicrobial resistance is a natural phenomenon that occurs when microorganisms no longer respond to antibiotics to which they were previously susceptible and that were previously active in treating infections caused by these microorganisms [10,12]. As a result of drug resistance, infections become harder or impossible to treat, increasing the risk of the spread of serious infectious diseases and death [13,14]. The spread of AMR as a process caused by the overuse of antibiotics is an unfulfilling definition since it has long been known that AMR occurs naturally over time through distinct mechanisms [15,16]. In other words, the excessive use of antibiotics in both humans and animals results in an acceleration of this natural process, thus promoting the spread of AMR [17,18]. We often refer to bacteria becoming resistant to antibiotics but we very rarely think about what this means. Within this contest, it is possible to distinguish two types of resistance: natural, which can be further categorized into intrinsic and induced, and acquired [19]. The intrinsic resistance is when bacterial species are naturally resistant to certain classes of antibiotics and obviously it is independent of previous antibiotic exposure (e.g., vancomycin resistance in *Escherichia coli* and ampicillin, 1st and 2nd generation cephalosporins resistance in *Pseudomonas aeruginosa* [19,20]. Natural resistance in bacteria can also be induced by the activation of genes as a result of exposure to clinical amounts of antibiotics [21]. The acquired resistance can occur through two distinct processes: a mutation that occur in the DNA of the cell during the replication or DNA transfer (Figure 1). Regarding the first way, the mutant strains are capable of transferring the mutation to the progeny via the vertical pathway [19,22]. The second way through which bacteria acquire resistance is through transformation, transposition and conjugation (all termed horizontal gene transfer) (Figure 1). In transformation, the recipient bacterium takes up extracellular donor DNA. In transduction, donor DNA packaged in a bacteriophage infects the recipient bacterium. In conjugation, the donor bacterium transfers DNA to the recipient by mating.

Antibiotic-resistant genetic material is then transferred from the antibiotic-resistant bacteria to the non-resistant bacteria that become resistant to antibiotics [23].

## 3. How Bacteria Acquire Resistance

**Figure 2:**
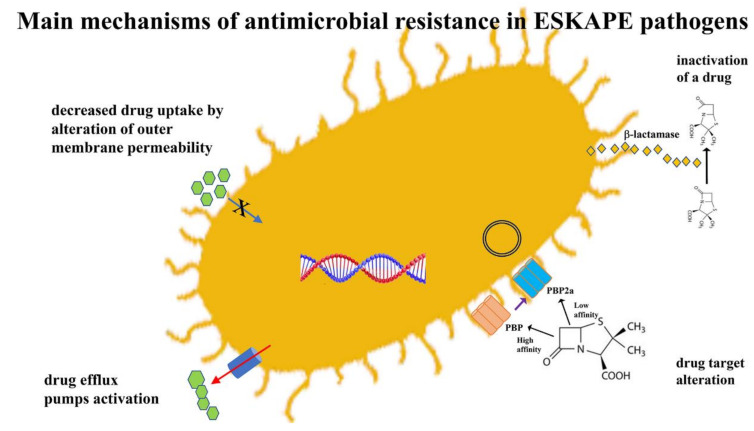
Mechanisms of antibiotic resistance in ESKAPE pathogens.

The rapid spread of AMR through bacterial populations cannot be attributed to a single mechanism. It is often the result of complex processes. It is therefore necessary to subdivide antibiotics into groups based on the different mechanism of action before analyzing the factors that affect resistance to these molecules. Although there are many different classes of antibiotics, in this review, we have chosen to describe those most closely involved in the occurrence of antibiotic resistance. Table 1 summarizes the mechanisms of action and resistance of the main groups of antibiotics. The main mechanisms of action of antimicrobial agents, detailed in Table 1, involve the inhibition of several bacterial processes that are involved in the synthesis of the cell wall, proteins, nucleic acids and the inhibition of metabolic pathways. The main mechanisms of resistance are: decreased drug uptake, drug target alteration, drug inactivation and drug efflux pumps activation [19,24] (Figure 2).

Since the mechanism of action of different antibiotics is largely dependent on both the nature of their structure and the affinity of these agents for different bacterial structures, it follows that knowledge of the mechanism of action of these agents is “the condition sine qua non” for understanding the emergence of resistance to these drugs [25,26]. A description of the most commonly-used classes of antibacterial drugs is available as Supplementary Materials.

In this review, the resistance of bacterial pathogens is discussed according to their categories established by the WHO [12]. 

## 4. The Main Difficult-to-Treat Antibiotic-Resistant Pathogens

### 4.1. Acinetobacter baumannii

Acinetobacter baumannii is an aerobic gram-negative bacillus that belongs to the group of pathogens grouped under the acronym “ESKAPE” (Enterococcus faecium, Staphylococcus aureus, Klebsiella pneumonia, Acinetobacter baumannii, Pseudomonas aeruginosa, and Enterobacter species), which refers to the ability of these bacteria to escape the effect of bactericidal activity of antibiotics [6,27]. *A. baumannii* is an opportunistic pathogen that causes hospital-acquired infections worldwide and can develop resistance to antibiotics by different mechanisms such as:

(1) the production of enzymes that degrade beta-lactam antibiotics. The production of all four classes of β-lactamases (A, B, C, and D) through the incorporation of exogenous DNA into its genome would underlie the rapid evolution of this strain toward multi-resistance [28,29]. Moreover, in *Acinetobacter* spp. have been identified both the genes encoding for narrow-spectrum β-lactamases (i.e., TEM-1, SCO-1, and CARB-4) and those encoding for ESBL (GES-11 and CTX-M) [29,30]. As stated above, class B β-lactamases are metallo-β-lactamases (MBLs) that have a broad substrate range, being able to inhibit all β-lactam antibiotics except the monobactams [31]. Class C β-lactamases are a group of broadly disseminated enzymes usually resistant to cephamycins (cefoxitin and cefotetan), penicillins and cephalosporins [32,33]. *A. baumannii* also possesses Class D or OXAs β-lactamases that can hydrolyze extended-spectrum cephalosporins and carbapenems [33,34]. Moreover, *A. baumannii* has an intrinsic *ampC* cephalosporinase [35];
(2) the expression of efflux pumps. In *A. baumannii* efflux pumps are involved in bacterial resistance to a number of antibiotics belonging to different chemical classes such as aminoglycosides, tetracyclines, erythromycin, chloramphenicol, trimethoprim, fluoroquinolones and different beta-lactams [36,37]. Different studies have shown that at least four classes of efflux pumps are associated with *A. baumannii* antimicrobial resistance: the major facilitator superfamily (MFS), the resistance nodulation division (RND) superfamily, the multidrug and toxic compound extrusion (MATE) family and the small multidrug resistance (SMR) family transporters [36,38]. More recently, an overexpression of the Ade ABC efflux pump, a member of the RND, was associated with tigecycline resistance in *A. baumannii* [39];
(3) the enzymatic modification of aminoglycosides. Enzymatic modification is the most common type of aminoglycoside resistance [40]. Acetyltransferases, adenylyltransferases and phosphotransferases are three classes of enzymes that play a critical role in the resistance of *A. baumannii* to aminoglycosides [41]. The genes encoding for aminoglycoside modifying enzymes can be transferred through plasmids and transposons [41].
(4) the production of modified porins that decreases the permeability of the outer membrane [42,43]. In *A. baumannii* the reduced expression of porins, proteins that allow the transport of molecules across the outer membrane, is associated with carbapenem resistance [29,44]. Moreover, *A. baumannii* may acquire resistance to colistin, a polypeptide antibacterial agent that targets LPS, as a result of mutation of the genes involved in LPS biosynthesis [45,46];
(5) the modification of the antibiotic target [47]. In *A. baumannii* this mechanism of resistance is mediated by overexpression of penicillin-binding proteins that results in imipenem resistance or by mutations of DNA gyrase that prompts quinolone and tetracycline resistance [29,30].

Until a few years ago, carbapenems like imipenem and meropenem were the most effective agents to treat *A. baumannii* infections [48]. These agents were replaced by minocycline/tigecycline until the resistance of this microorganism to these two agents also became significant [48,49]. Ampicillin + sulbactam + carbapenem combination is the best therapy for treating MDR *A. baumannii* bacteremia [50]. Minocycline therapy is also effective, although significant rates of resistance has been recorded [48]. Minocycline-resistant *A. baumannii* infections are treated with a combination of minocycline and colistin while colistin/rifampin is the most effective treatment for colistin-resistant *A. baumannii* [51]. Moreover, trimethoprim-sulfamethoxazole combined with colistin rapidly kills carbapenem-resistant *A. baumannii* [52,53]. However, strains resistant to these antibiotics are often isolated as well. From the above, it is evident that every effort must be done to find out new antibiotics capable of killing MDR *A. baumannii*.

### 4.2. Pseudomonas aeruginosa

*P. aeruginosa* is an aerobic gram-negative bacterium commonly found in the environment and one of the most common pathogens responsible for a variety of acute and chronic nosocomial infections including severe respiratory infections in patients with compromised host defenses [54,55]. In this context, *P. aeruginosa* is the third most common gram-negative bacteria causing nosocomial bloodstream infections [56]. *P. aeruginosa* has shown intrinsic resistance to many antibiotics that is due to different mechanisms of resistance that are both intrinsic and acquired from other microorganisms [57,58]. The main mechanisms of resistance are: over-expression of efflux pumps, decreasing outer membrane permeability and acquisition or mutation of resistance genes that encode for proteins that control the passive diffusion of antibiotics across the outer membrane [59,60]. Ceftazidime and cefepime belonging, respectively, to the third and fourth generation of cephalosporins, are broad-spectrum antimicrobials that have *P. aeruginosa* coverage [61]. Like *A. baumanni*, also in *P. aeruginosa* all four major classes of β-lactamases (A, B, C and D) have been identified [62]. Endogenous β-lactamase such as AmpC β-lactamase can be induced by several β-lactams such as benzylpenicillin and imipenem [63]. Moreover, *P. aeruginosa* can acquire resistance through a gene mutation which leads to overexpression of AmpC β-lactamases [64]. *Pseudomonas* resistance to aminoglycosides is mediated by transferable aminoglycoside modifying enzymes (AMEs) that decrease the binding affinity to their target in the bacterial cell [65,66]. The treatment of MDR *P. aeruginosa* involves colistin in combination with an anti-pseudomonas agent like imipenem, piperacillin, aztreonam, ceftazidime or ciprofloxacin [66,67]. Drug resistance in *P. aeruginosa* have been successfully treated with fosfomycin in combination with aminoglycosides, cephalosporins and penicillins [63,66]. 

### 4.3. Staphylococcus aureus

*S. aureus*, a major human pathogen, is a gram-positive, facultative anaerobe, catalase- and coagulase-positive coccus that tends to form irregular grape-like clusters [68]. *S. aureus* causes infections ranging from mild to life-threatening such as skin and soft tissue infections, bacterial endocarditis, pleuropulmonary and device-related infections [69]. This microorganism is an important human pathogen not only because it is highly contagious and capable of inducing long-lasting chronic infections but also because of its great ability to develop resistance against old and new antibiotics [70]. For example, about three years after the discovery of penicillin appeared penicillin-resistant *S. aureus* carrying a plasmid-encoded beta-lactamases capable of hydrolyzing the β-lactam ring of penicillin [71]. This gene is carried on transposable elements that have moved into plasmids which often also carried genes resistant to other antibiotics such as erythromycin and gentamicin [71,72]. In 1959, methicillin, a semi-synthetic penicillin, was introduced to combat infections caused by penicillin-resistant bacteria; however, as early as 1961 the first methicillin-resistant *S. aureus* strain was identified [72,73]. Methicillin and other β-lactam antibiotics inhibit the growth of *S. aureus* by binding to the penicillin-binding proteins (PBPs). *S. aureus* became resistant to methicillin (MRSA) by acquiring, via horizontal gene transfer, the genes *mecA* and *mecC* which inactivate methicillin by the synthesis of an alternative PBP, designated PBP2a, that has very low affinity for almost all β-lactam antibiotics [73,74]. For many years vancomycin has been considered a last-resort antibiotic against severe MRSA and other resistant gram-positive infections [75]. However, by the late 1980s vancomycin resistance first appeared in enterococci (VRE) and in recent years in *S. aureus* (VRSA) [76]. The resistance mechanism of VRSA is mediated by the *VanA* operon carried on the mobile genetic element Tn1546 acquired from vancomycin-resistant *Enterococcus* [77,78]. In 1997, reported for the first time was the first clinical isolate of vancomycin-intermediate *S. aureus* (VISA) which is not inhibited in vitro at vancomycin concentration below 4–8 µg/mL. In contrast, vancomycin-resistant *S. aureus* (VRSA) is inhibited only at concentrations of 16 µg/mL or more [77]. VISA and VRSA have emerged from MRSA; however, VRSA does not progress from VISA because both have different resistance mechanisms [79].

According to the World Health Organization (WHO), the pathogenicity and antibiotic resistance pattern of *S. aureus* poses a severe threat to human health worldwide [10]. MRSA, VISA and VRSA are well-recognized as a major pathogen of hospital acquired infections and are considered to be high priority agents since, without effective containment and therapeutic solutions, they could cause serious infections that are impossible to control worldwide [80]. MRSA infections are usually difficult to treat, and thus several classes of antibiotics have been used over the past decade to treat these infections that have contributed to the emergence and spread of MDR strains [71,80,81]. In MRSA the resistance to a single antimicrobial agent as well as to different classes of antibiotics occurs through the activation of several different mechanisms such as (1) mutation in target genes (e.g. the resistance towards fluoroquinolones is due to mutation in *gyrA* and *gyrB* genes of topoisomerse II); (2) target alterations; (3) overexpression of efflux pump (NorA pump) [71]. Daptomycin, a cyclic peptide antibiotic with a fatty acid side chain that bind to the bacterial cytoplasmic membrane in the presence of calcium ions, is an important alternative to vancomycin for the treatment of patients with infections caused by MRSA [82]. However, although daptomycin resistance in *S. aureus* is uncommon, resistance to this drug during therapy is increasing due to mutations of different proteins that result in a reduced drug binding to its target site [28,29]. Moreover, *S. aureus* is well known for its ability to acquire resistance to other antibiotics such as trimethoprim-sulphamethoxazole and tetracyclines by aforementioned different mechanisms of resistance [72]. Since high resistance rates were noted in patients who received prolonged courses of fusidic acid or rifampicin monotherapy a combination therapy is a rational option for *S. aureus* skin infections [71,83]. In recent years, due to the increasing rate of MRSA infection, there is a renewed interest in the use of macrolide-lincosamide-streptogramin (MLS) agents to treat such infections [71,84]. Given the excellent pharmacokinetic properties (i.e., clearance, elimination half-life, large tissue penetration) of clindamycin, this lincosamide antibiotic is the most favored agent for the treatment of serious infections, including those caused by macrolide resistant *S. aureus* and MRSA. However, numerous reports indicate that clindamycin resistance is also increasing among health care-associated MRSA strains. MLS resistance is due to three main mechanisms: target modification, active efflux and enzymatic antibiotic inactivation [85]. Among them the ribosomal target modification mechanism mediated by *erm genes* (*ermA*, *ermB*, *ermC* and *ermF)* is the main mechanism [85]. These genes encode for methyltransferases that modify the ribosomal target site locking the binding of the antibiotic and conferring constitutive and inducible resistance [86]. Inducible resistance is developed when a suitable macrolide inducer (e.g. erythromycin), of the methyltransferases, is present. These strains are resistant to erythromycin and falsely susceptible to clindamycin in vitro. [86]. However, if the strain is resistant to erythromycin it is possible that during clindamycin therapy may be selected mutants resistant to clindamycin and patients may not respond clinically to clindamycin because of a modification of the ribosomal target. Inducible clindamycin resistance can be detected by standard automated susceptibility testing devices or alternatively must be detected by the double-disk diffusion test (D-test) [86,87]. Infections caused by a MRSA strain with a positive D-test should not be treated with clindamycin [73].

### 4.4. Klebsiella pneumonia

*K. pneumoniae* is a member of the family Enterobacterales, non-fastidious, commonly encapsulated gram-negative bacillus [8]. *K. pneumoniae* can cause different types of nosocomial and community acquired infections, including urinary tract infections, pneumonia, liver abscess, surgical site infections and bloodstream infections especially in immunocompromised patients [88,89]. Since the bacteria doesn’t spread through the air, to get a *Klebsiella* infection person-to-person contact is required [90]. *Klebsiella* has become highly resistant to antibiotics by the widespread acquisition of genes encoding enzymes, such as ESBLs and carbapenemases [91]. Carbapenem-resistant *K. pneumoniae* strains are the most clinically prominent carbapenem-resistant Enterobacteriaceae (CRE) [92]. Carbapenems often are the last line of defense against gram-negative persistent infections, therefore the increasing prevalence of carbapenemase-producing *K.* *pneumoniae* (*KPC*) strains harboring the carbapenemase encoding *blaKPC-3* gene, is a major threat to public health [93,94].

### 4.5. Enterobacter Spp.

*Enterobacter* species are motile aerobic gram-negative bacilli belonging to Enterobacteriaceae family. The *Enterobacter cloacae complex* (ECC) includes different pathogens, capable of producing a wide variety of infections, the most frequent of which are *Enterobacter cloacae* and *Enterobacter aerogenes* [95]. In 2019, *E. aerogenes* was re-classified as *Klebsiella aerogenes* owing to its higher genotypic similarity with the genus *Klebsiella* [96]. *Enterobacter* species are non-fastidious gram-negative rods that are sometimes encapsulated [97]. They can cause opportunistic infections in immunocompromised, usually hospitalized, patients having acquired a wide range of antibiotic resistance mechanisms [96]. Many *Enterobacter* strains produce ESBLs and carbapenemases, including VIM, OXA, metallo-β-lactamase-1, and KPC [34]. Furthermore, in this bacterial group, an important role in the development of antibiotic resistance is represented by the permanent depression of ampC β-lactamases, which can be expressed at high levels. [98]. These MDR strains are resistant to almost all available antimicrobial drugs, except tigecycline and colistin [8,99]. Moreover, a recent report indicates that pan-drug-resistant *K. aerogenes* has also emerged, displaying resistance to the last-resort antibiotic colistin [7]. To further complicate the treatment of bacterial infections, *K. aerogenes* is capable of harboring subpopulations of colistin-resistant bacteria which are undetectable using current diagnostic testing strategies [100].

### 4.6. Enterococci

Enterococci are gram-positive cocci, facultative anaerobes gastrointestinal commensals capable of persisting in a range of stressful and hostile environments [101]. Although more than 50 different species of enterococci have been described, only two species in human cause the majority of enterococcal infections: *E. faecalis* and *E. faecium* [101]. *E. faecalis* is the most pathogenic species although *E. faecium* is more resistant to many antimicrobial agents and especially in immunocompromised hosts the latter can cause severe morbidity and mortality [101,102]. In general, these microorganisms are typically harmless in healthy individuals while in immunocompromised patients are involved in hospital-acquired infections such as catheter-associated urinary tract infections, endocarditis and bacteremia [102]. Enterococci are becoming increasingly resistant to antimicrobial agents and this is mainly due to: (1) the large use in hospitals of broad-spectrum antibiotics (penicillins and cephalosporins) promotes intestinal colonization of *E. faecium* by greatly increasing the normal gram-negative intestinal microbiota (mutated PBP and the overexpression of β-lactamase enzymes lead to high levels of resistance to β-lactam antibiotics) [103]; (2) the intrinsic resistance of enterococci to several commonly used antibiotics [104]; (3) the capacity of these strains to acquire and disseminate determinants of antibiotic resistance [104]. In *E. faecium,* at least three different pathways involved in cephalosporins resistance have been identified [103,104]. In the 1970s, vancomycin was introduced to contrast the diffusion of enterococci resistant to third-generation cephalosporins [105]. Then, in the 1990s due to the heavy use of vancomycin, vancomycin-resistant enterococci (VRE) emerged as the second most common nosocomial pathogen [104,105]. *E. faecium* can acquire genes through mobile genetic elements such as plasmids and transposons (i.e vancomycin resistance can be transferred by the *vanA* gene cluster on the transposon Tn*1546*) [106]. Vancomycin acts by targeting the D-alanyl-D-alanine terminus of peptidoglycan inhibiting cell wall synthesis [107]. Vancomycin-resistance is mediated by several *van* gene clusters such as *vanR*, *vanS*, *vanH*, *vanX* and *vanZ* that are responsible for the replacement of D-Ala-D-Ala with D-alanyl-D-lactate termini. Vancomycin binds to d-Ala–d-Lac much more weakly than it does to the normal dipeptide product resulting in a low binding affinity of vancomycin [106]. *Van A* gene cluster is the most common type and was located on transposon on a 10,581-bp transposon (Tn*1546*) of *E. faecium* [108].

*E. faecium* is considered a MDR bacteria since it is intrinsically resistant to aminoglycoside like tobramycin, kanamycin, gentamicin being capable of producing aminoglycoside-modifying enzymes (AMEs) including aminoglycoside nucleotidyltransferases (ANTs) aminoglycoside acetyltransferases (AACs) and aminoglycoside phosphotransferases (APHs) [109]. Moreover, mutations within the *rpsL* gene, which encodes the ribosomal protein S12, can result in high level resistance to streptomycin [106,109]. Moreover, high-level fluoroquinolones resistance in *E. faecium* is most frequently linked with point mutations in *gyrA* and *parC* genes that encode subunits A of DNA gyrase and topoisomerase IV or with efflux transporter *NorA* that pump out these drugs [106]. 

## 5. Conclusions

Antibiotic resistance is the ability of bacteria to resist exposure to antibiotics designed to kill them or inhibit their growth. Although antibiotic resistance is a natural process due to genetic changes in the bacteria following antibiotics exposure, however, this phenomenon is being accelerated through the overuse and misuse of antibiotics. Overuse of antibiotics causes susceptible bacteria to be killed and allows drug-resistant bacteria to proliferate. Poor sanitation, poor infection control and the use of antibiotics in farm animals are among the main reasons for the spread of antimicrobial resistance. In addition, there are novel and often underrecognized mechanisms of resistance that further contribute to drug resistance such as the heteroresistance (HR) and the mutant prevention concentration (MPC). The first of these two factors can be defined as resistance to certain antibiotics by a preexisting subpopulation of resistant cells, within a larger population of antimicrobial-susceptible microorganisms [110]. This sub-population of resistant cells can rapidly replicate in the presence of a given antibiotic whereas the susceptible microorganisms are killed. Recent reports indicate that heteroresistance is very common for several bacterial species and classes of antibiotics [110]. The second one, known as MPC, represents a threshold above which the selective proliferation of resistant mutants is expected to occur only rarely [111]. Traditionally, the minimum inhibitory concentration (MIC) has been widely used to determine the susceptibility and resistance of bacteria to antimicrobials. However, MIC represents one parameter of resistance, but not all. Due to spontaneous mutations, even after exposure of cells to an antibiotic at MIC levels, a subpopulation of antibiotic-resistant mutants often remains. Increasing the concentration of the antibiotic above the MIC will result in a value that will kill all mutants [112]. This concentration is the MPC that can be defined as the MIC of the least-susceptible, single-step mutant. In this context, it is essential to determine the MPC/MIC ratio in order to prevent the emergence of mutant. 

The ESKAPE pathogens represent deadly bacteria with rapidly growing multi-drug resistant properties. Although these bacteria are genetically different, the resistance strategies that underlie the emergence and persistence of these pathogens are widely shared among them including decreased drug uptake, drug target alteration, drug inactivation and drug efflux pumps activation. To limit the spread of ESKAPE pathogens and antibiotic resistance more generally, it has become imperative to be more careful in surveillance and implementation of antimicrobial stewardship in both human health and food animals. Implementation of these programs together with the development of new antibiotics or new approaches (e.g. inhibition of biofilm formation and bacteriophage therapy) are likely the only way to slow the spread of multi-drug resistant strains worldwide.

## Figures and Tables

**Table 1:** Mode of action and resistance mechanisms of antibiotics.

Antimicrobial Groups	Mechanism of Action	Resistance Mechanism
β-Lactams Penicillins	Inhibits cell wall production	Beta-lactamase production Penicillinase
Cephalosporins Carbapenems		Cephalosporinase Carbapenemase
β-Lactamase inhibitors	Block the activity of beta-lactamase enzymes	Extended-spectrum beta-lactamase (ESBL)
Aminoglycosides, Chloramphenicol Macrolides, Tetracyclines	Inhibit ribosome assembly by binding to the bacterial 30S or 50S (inhibit protein synthesis)	Multifactorial (enzymatic modification, target site modification and efflux pumps)
Fluoroquinolone	Inhibit DNA replication	Multifactorial (target-site gene mutations, efflux pumps and modifying enzyme)
Sulfonamides and trimethoprim	Inhibit folic acid metabolism	Horizontal spread of resistance genes, mediated by transposons and plasmids, expressing drug-insensitive variants of the target enzymes.

## Supplementary Materials

The following are available online at https://www.mdpi.com/article/10.3390/pathogens10101310/s1, Supplementary Materials.
