# Supplementary material for: Bacterial Antibiotic Resistance: The Most Critical Pathogens
Source: Pathogens. 2021 Oct 12;10(10):1310. doi: 10.3390/pathogens10101310 (PMC8541462; doi:10.3390/pathogens10101310)
Supplement: Supplementary file 1 [file pathogens-10-01310-s001.zip › pathogens-1408896-supplementary.pdf]

pathogens

MDPI

# Supplementary materials

## 1.β-. lactam antibiotics: mechanisms of action and resistance

β-lactam antibiotics are one of the oldest and widest classes of antibiotics characterized by the presence of a beta-lactam ring in their molecular structure [1]. All beta-lactam antibiotics such as penicillin, cephalosporin, and carbapenem are bactericidal and act by inhibiting the synthesis of bacterial peptidoglycan cell walls [2]. These antibiotics act on enzymes called penicillin-binding proteins (PBPs) involved in the cross-linking of the bacterial cell walls. Specifically, the beta lactam ring portion of these antibiotics binds irreversibly to PBPs by inhibiting the crosslinking of peptidoglycan thus triggering the death of the bacteria by autolysis [3]. The basic structure of the penicillins is 6- aminopenicillanic acid (6-APA), otherwise known as the β-lactam ring nucleus, that in penicillins, cephalosporins and carbapenems, is fused to another 5- or 6- member ring, whereas in monobactams, the β-lactam ring is monocyclic [4]. The positions 1, 2, 3, or 4 of the β-lactam ring have been evaluated for the introduction of numerous and different substituent groups in order to improve the biological activity of penicillins (e.g., the methicillin was the first semisynthetic penicillin able to resist hydrolysis by the penicillinase that was approved for clinical use in the United States in 1960) [4,5]. Moreover, the carbenicillin, a semi-synthetic compound effective against *P. aeruginosa*, with a carboxyl group in place of amino group of ampicillin, was introduced in 1967 [6]. Ampicillin and amoxicillin are aminopenicillins antibiotics of the penicillin family active against gram-positive and some gram negative bacteria. The amine group increases the rate of entry into the Gram-negative bacteria and stability togastric juices [7]. To broaden their antibacterial spectrum of activity, ampicillin and amoxicillin are often given in combination with beta-lactamase inhibitors, sulbactam and clavulanic acid, respectively [8]. Beta-lactam antibiotics do not have the same efficacy against all types of bacteria and this depends mainly on the structural characteristics that some bacterial species possess that hinder the activity of these antibiotics (for example, the outer membrane of Gram-negative bacteria does not favor the interaction of these antibiotics with the PBPs) [9,10]. An important broad-spectrum group of antibiotics closely related to the penicillins are the cephalosporins originally derived from the mold *Acremonium* (previously known as "Cephalosporium"). Cephalosporin C (CPC) produced by *Acremonium chrysogenum* is one of the most important antimicrobials for treatment of bacterial infections [11]. This compound generated an entirely new family of β-lactam antibiotics because instead of 6-APA, it possesses a nucleus of 7-aminocephalosporinic acid. Using 7-ACA as generations of cephalosporins with potent broad-spectrum activity have been synthesized [12]. Semi-synthetic cephalosporins are produced by modifying the side chains linked to the nucleus and are commonly grouped into fifth generations based on their antimicrobial properties [12]. The main difference between the five different generations lies in different ways of administration and the higher activity against Gram-negative bacteria of one generation compared to the previous one. Of particular note, the fourth-generation cephalosporins are more effective against Gram-negative bacteria compared to the first-generation cephalosporins and the broad-spectrum activity of fifth generation cephalosporins against Gram-positive bacteria, MRSA and penicillin-resistant pneumococci [13].

Carbapenems are often used as “last-line agents” to treat 51 infections due to resistant bacteria [14]. They have a penicillin-like five-membered ring but it differs from that of penicillin in that it is unsaturated and contains a carbon atom rather instead of sulfur [15]. The structural similarity of the carbapenems to the terminal amino acid residues of the peptidoglycan (acylated D-alanyl-D-alanine) allows carbapenems to bind irreversibly to the active site of the PBPs, leading to the inhibition of transpeptidation that in turn disrupts the cell wall synthesis [16].

The first naturally discovered carbapenem was the thienamycin, a derivative produced by *Streptomyces cattleya* [14]. The unique chemical structure of carbapenems makes them resistant to the action of the majority of β-lactamases, including extended spectrum β-lactamases (ESBLs). However, because of its instability in water the thienamycin in clinical therapy is used as N-formimidoyl thienamycin, a semisynthetic derivative known as imipenem [17,18]. Imipenem is co-administered with cilastatin, which has the dual function of inhibiting dehydropeptidase I, a renal enzyme that can degrade the imipenem and protecting against imipenem-induced nephrotoxicity. [19,20].

Carbapenems such as imipenem/cilastatin, meropenem, doripenem and ertapenem are the latest developed β-lactams possessing a broad spectrum of activity and are usually reserved for treating infections caused by multidrug resistant (MDR) pathogens [15,21]. However, ertapenem effectiveness against non-fermentative rod-shaped bacteria like *Pseudomonas aeruginosa* is relatively limited when compared with meropenem or imipenem [22]. Imipenem/cilastatin is effective for the treatment of a wide variety of bacterial infections, including complicated urinary tract infections, lower respiratory tract infections and, most importantly, for the treatment of infections caused by cephalosporin-resistant nosocomial bacteria [15,23].

Three major mechanisms are involved in the resistance to carbapenems: porin loss, efflux pumps and acquisition of carbapenemase genes [15,24]. Since the porins control the passive diffusion of antibiotics across the outer membrane (OM), the deficiency of porin expression results in a decreased absorption of antibiotics (i.e. a reduced expression of ompk35 and ompk36, two trimeric porins of *Klebsiella pneumoniae*, results in increased resistance of strains that harboring these mutations to ertapenem) [25,26].

Overproduction of efflux pumps is an efficient mechanism for the carbapenem resistance since these pumps can expel outside the cells many different antimicrobial agents [27]. Carbapenem resistance in *P. aeruginosa* is either mediated via a combination of efflux pumps, AmpC overexpression and porin loss [28]. The over production of several efflux pumps is considered to play an important role in low-level resistance to various carbapenems in *A. baumannii* [29]. The third mechanism dealing with carbapenem resistance arises from the acquisition of carbapenemase encoding genes capable of hydrolyzing carbapenems and other β-lactam antimicrobials [30]. Since these enzymes are encoded by genes carried on transposable elements or plasmids can be horizontally transferred to other bacterial species, making this resistance mechanism the greatest threat [31]. Carbapenemases are β-lactamases that based on their molecular structure belong to Ambler molecular classes A, B, C, and D. Classes A, C, and D inactivate the β-lactams through the catalytic activity of serine-residue, whereas class B or MBLs need zinc cation for their action [22,32].

*β-lactamases* have been also classified based on the functional classification of Bush-Jacobi-Medeiros into groups 1 to 3 depending on the degradation of β-lactam substrates and the effects of inhibitors [32,33]. Ambler Classes A and D are known as serine β-lactamases (SBLs) and utilize serine for β-lactam hydrolysis [34]. Ambler Class B are known as metallo-β-lactamases (MBLs), since require divalent zinc ions for substrate hydrolysis [32,34]. Clavulanic acid, sulbactam and/or tazobactam, known β-lactamase inhibitors, can inhibit SBLs but are MBLs ineffective [35]. The latter may be inhibited by metal ion chelators, such as dipicolinic acid or EDTA that however are not approved for clinical use [36].

Class A β-lactamases include: Principal component 1 (PC1), a common source of penicillin resistance in *S. aureus [37]*; TEM, the designation *TEM* refers to *Temoneira,* a Greek *patient* from whom was isolated an *E. coli* carrying the *TEM*-1 β-lactamase and active against aminopenicillins and early cephalosporins [38]; SHV (sulfhydryl variant of TEM), originally detected on the chromosome of *K. pneumoniae* and subsequently by mechanisms of mobilization between different bacteria have been carried to plasmids *[34,39]*; CTX-M (cefotaximase), a group of class A β-lactamases rapidly spreading among Enterobacteriaceae worldwide [40]; and KPC (*K. pneumoniae* carbapenemase) [41]. TEM, SHV and CTX-M families being plasmid and transposon mediated, have been quickly spreaded to different species particularly between the Enterobacteriaceae [42]. Moreover, acquisition of

point mutations has allowed TEM and SHV to acquire the ability to hydrolyze cephalosporins such as cefotaxime and ceftazidime thereby generating the so called "extended-spectrum" phenotype (extended-spectrum beta-lactamases, or ESBLs) [43]. Like TEM and SHV, CTX-M enzymes, also have been affected by point mutations that enlarged their activity and provide resistance to different β-lactams [44]. ESBLs are becoming a major threat for the effectiveness of cephalosporins in different clinical contexts worldwide [45].

As mentioned above, carbapenemases belong to classes A, B and D of β- lactamases. Class A carbapenemases include six enzymes: NmcA (not metalloenzyme carbapenemase A), SME (*Serratia marcescens* enzyme), IMI-1 (Imipenem-hydrolysing β-lactamase), and SFC-1 (*Serratia fonticola* carbapenemase-1); whereas these four enzymes are chromosomally encoded, KPC (KPC-2 to KPC-13), so named because it was first identified in 1996 in *K. pneumoniae*, and derivatives of GES (Guiana extended spectrum, GES- 1 to GES-20) are plasmid encoded [46–48]. A few years after their discovery, KPC-producing strains have spread worldwide, partly due to the fact that they are usually multidrug resistant (i.e. resistant aminoglycosides, fluoroquinolones and trimethoprim-sulfamethoxazole) [49]. KPC enzymes initially identified in *K. pneumoniae*, more recently, have been found also in *P. aeruginosa* and *A. baumannii* [50].

Class B β-lactamase (MBLs) are mainly plasmid-encoded enzymes that can inactivate the majority of β-lactams, with the exception of monobactams [51]. Their activity is inhibited by by EDTA, a chelator of divalent cations [34,51]. These enzymes include the New Delhi MBL (NDM), Imipenem-resistant *Pseudomonas* (IMP)-type carbapenemases, VIM (Verona integron-encoded MBL), GIM (German imipenemase) and SIM (Seoul imipenemase) [52,53]. New Delhi MBL (NDM) is an MBL that confer resistance to β-lactams, including carbapenems but not to aztreonam [54]. Currently, up to 8 variants have been identified both in enteric pathogens such as *K. pneumoniae*, *E. coli* and other gram-negative bacteria such as *A. baumannii* and *P. aeruginosa* [22,55,56]. IMP type carbapenemases have been identified for the first time in 1991 in Japan and since then up to 18 variants have been identified worldwide [57]. It is worth mentioning that the majority of these enzymes were investigated in *Acinetobacter* spp, *Pseudomonas spp* and enteric pathogens [47,58]. VIM-1 (Verona integron-1- encoded metallo β-lactamase) was first described in a *P. aeruginosa* isolate in Verona, Italy, with more than 10 variants being identified thereafter [59,60]. VIM, like other members of this class, are able to hydrolyzing most β-lactams while are susceptible to all β-lactam inhibitors but not to aztreonam [61]. However, although MBLs are not capable of recognizing and inactivating monobactams it also true that these enzymes are often co-expressed with SBLs that, unlike MBLs, are able to hydrolyze these antibiotics [51,62].

*AmpC* β-lactamases are clinically important cephalosporinases that belong to Ambler class C or to group 1 based on respectively, molecular structure or functional classification [63,64]. *ampC* β-lactamase genes are encoded on the chromosomes of numerous *Enterobacteriaceae. E. coli* naturally carries *a chromosome-mediated ampC* gene but it is not inducible and hence it is possible to treat *E. coli* infections with β-lactam antibiotics [64–66]. Although *ampC* β- lactamase genes are encoded on the chromosomes of numerous *Enterobacteriaceae* they can be also plasmid-encoded (plasmid-mediated ampC, pampC) [67]. *pampC* genes can be spread by horizontal gene transfer as documented in different strains lacking inducible *ampC* genes such as *Klebsiella* spp., *Proteus mirabilis*, *Salmonella enterica* and *Shigella spp* [64,68,69]. Moreover, in many gram-negative bacteria including *Enterobacter spp., Citrobacter freundii, Serratia marcescens, Morganella morganii and P. aeruginosa*, induction of *ampC* gene involves other genes products such as AmpR, AmpD, AmpG and AmpE that encode, respectively, for a positive transcriptional regulator, a transmembrane protein that acts as a permease for 1,6-anhydromurapeptides, a cytosolic N acetyl-anhydromuramyl-L-alanine amidase and a cytoplasmic-membrane protein that acts as a sensory transducer molecule necessary for induction [11,64,70,71]. Inducible *ampC*-mediated β-lactam resistance is a problem especially in infections due to *E. aerogenes* and *E. cloacae* that initially to cephalosporins upon therapy become resistant to broad-spectrum agents including cefotaxime, ceftazidime and ceftriaxone [64]. Class D carbapenemases are also

known as "oxacillinases" because of their ability to hydrolyze oxacillin and carbapenems while have low hydrolysis activity toward cephalosporins [72,73]. Moreover, OXA β-lactamases are not inhibited by classical inhibitors such as clavulanate, sulbactam, and tazobactam, with some exceptions (i.e., OXA-2 and OXA-32 are inhibited by tazobactam) [74,75]. OXA-48 is one of the most frequent carbapenemases worldwide; it is not inhibitors and it is frequently detected in *K. pneumoniae* and *E. coli*, although can also occur in other enteric bacteria [76,77]. Variants of OXA-48, commonly referred to as OXA-48-like (i.e as OXA-23, and OXA-58) are frequently found in *Acinetobacter* spp and posed one of the greatest threats due to the lack of inhibitors for them [78,79]. Moreover, there are several OXA-48-like variants that change their spectrum of activity such as OXA-163, that has lost carbapenem activities and gained an ESBL phenotype [80,81].

## 2. Aminoglycosides: mode of action and mechanisms of bacterial resistance

The aminoglycosides are produced from strains of *Streptomyces* spp., *Micromonospora* spp., and *Bacillus* spp [82]. These antimicrobial compounds kill bacteria by binding to the bacterial ribosome 30S subunit and blocking the formation of the 70S ribosome [1]. They are particularly active against aerobic gram-negative bacteria and mycobacteria while they are ineffective against anaerobic strain (e.g., gentamicin need an oxygen-dependent active transport to pass through the gram-negative membrane) [83]. Aminoglycosides are also effective against gram-positive bacteria when used in combination with other types of antibiotics [84]. The principal aminoglycosides that are used clinically are neomycin, streptomycin, kanamycin, tobramycin and amikacin [85]. Bacterial resistance to aminoglycoside antibiotics occurs by three mechanisms: decreased cell permeability, alterations at the ribosomal binding sites or production of aminoglycoside modifying enzymes [85,86]. The latter is the main mechanism of aminoglycoside resistance and involves three families of enzymes: acetyltransferases (AAC), nucleotidyl (adenyl) transferase (ANT) and phosphotransferases (APH) [86].

## 3. Sulfonamides: mode of action and mechanisms of bacterial resistance

Sulfonamides are synthetic antibacterial compounds used in association with diaminopyrimidines (trimethoprim or ormetoprim) which together have a synergistic effect in inhibiting folic acid metabolism in bacteria [87]. Sulphonamides are structural analogs of para-aminobenzoic acid (PABA), and thus act as competitive antagonists with PABA for the enzyme dihydrofolate synthase, blocking the synthesis of dihydrofolic acid (DHFA) [88]. Trimethoprim inhibits dihyrofolate reductase, an enzyme that reduces dihydrofolic acid to tetrahydrofolic acid, locking the production of tetrahydrofolate to its active form of folate. When used alone these drugs are bacteriostatic while in combination these drugs inhibit two steps in the bacterial biosynthesis of nucleic acids and proteins with a bactericidal effect [88,89]. Toxicity is selective since mammalian cells unlike bacteria require preformed folic acid and cannot synthesize it [1]. Sulfamethoxazole-trimethoprim combination has been useful in the treatment of a variety of infections caused by gram-positive and gram-negative bacteria such as urinary tract infections and upper and lower respiratory tract infections [90,91]. While trimethoprim or sulphonamides resistance alone develops rapidly in bacteria, unlike the resistance to sulphonamides and trimethoprim in combinations occur more slowly [92]. Resistance occurs via chromosomal and plasmid-mediated mechanisms [1,92]. In particular, the main mechanism of sulfonamides resistance involves the mutations in *folP* gene encoding dihydropteroate synthase (DHPS) or through acquisition of alternative DHPS genes (*sul1*, *sul2*, and *sul3*), the products of which have low affinity to sulfonamides [93].

## 4. Fluoroquinolones: mode of action and mechanisms of bacterial resistance

The fluoroquinolones are synthetic broad-spectrum antibiotics such as: ciprofloxacin, gemifloxacin, levofloxacin, moxifloxacin and ofloxacin [94]. Fluoroquinolones directly inhibit bacterial DNA synthesis by the inhibition of two enzymes: topoisomerase II (DNA gyrase) and topoisomerase IV [94]. These two enzymes have a similar protein structure,

both being composed of four subunits (two A and two B) but different functions [1,95]. The principal function of DNA gyrase is catalyzes negative supercoiling of double-stranded closed-circular DNA. Topoisomerase IV has a critical role in the unlinking DNA after chromosomal duplication [96]. The inhibition of DNA gyrase generate gaps in the DNA strands that activate endonucleases, which initiate uncoordinated repair and irreversible damage of cells [96]. Fluoroquinolones have the same core quinolone structure and differ only for various chemical substitutions and side groups that account for variations in the lipophilicity, oral absorption, volume distribution, elimination rate and spectrum of activity (i.e.moxifloxacin has less activity against *P. aeruginosa* than ciprofloxacin) [96,97]. Fluoroquinolone resistance is usually chromosomally-mediated although plasmid-mediated, transferable fluoroquinolone resistance has been described [1,98]. The main mechanisms involved in fluoroquinolones resistance are linked to mutations quinolone-resistance determining regions (QRDRs), such as *gyrA* and *gyrB* in DNA gyrase and *parC* and *parE* in topoisomerase IV [99]. Moreover, active efflux pump can be overexpressed to enhance the excretion of quinolones from the cell. This enhanced efflux in turn causes increased minimum inhibitory concentrations of several drugs, including fluoroquinolones, tetracycline, chloramphenicol, and ampicillin [98,100]. Mutations that enhance efflux occur as a primary step to allow the bacteria to survive [100,101].

## 5. Tetracyclines: mode of action and mechanisms of bacterial resistance

Tetracycline antibiotics were isolated from various species of *Streptomyces* in the late 1940s [102]. Since the 1950s many semisynthetic structural modifications have been made on the tetracycline molecule to yield other tetracycline molecules with different pharmacokinetic properties and antimicrobial activities [102]. Tetracycline incorporates the related compounds oxy-, and chlortetracycline, doxycycline and minocycline [103]. The tetracyclines are a broad group of antibiotics that includes both those naturally produced (oxytetracycline and chlortetracycline, discovered in 1948) and second and third generation semi-synthetic derivatives such as doxycycline and minocycline [1,104]. Tetracyclines possess antibacterial activity by binding to the 30S ribosomal subunit of a susceptible organism [104]. Following ribosomal binding, the tetracycline interferes with the binding of aminoacyl-tRNA to the messenger RNA molecule/ribosome complex, thus disrupting the bacterial protein synthesis [105]. Tetracycline binds with the 70S ribosomes found in mitochondria and can also inhibit protein synthesis in mitochondria [1]. Tetracyclines are bacteriostatic and they are effective against a wide variety of gram-positive and gram-negative multiplying bacteria. Resistance to tetracycline is due to acquisition of resistance *tet* genes, carried in plasmids and transposons and transmitted through conjugation, coding for proteins that activate diverse mechanisms such as tetracycline efflux mediated by membrane efflux proteins and ribosomal protection mediated by altered target whereby the ribosome is protected from binding of tetracyclines [105–107]. A third, less common mechanism involves the synthesis of bacterial enzymes that attack the tetracycline [106].

## References


1. Kaufman, G. Antibiotics: mode of action and mechanisms of resistance. *Nursing standard* **2011**, *25*, 49-55, doi:10.7748/ns.25.42.49.s52.
2. Bush, K.; Bradford, P.A. beta-Lactams and beta-Lactamase Inhibitors: An Overview. *Cold Spring Harbor perspectives in medicine* **2016**, *6*, doi:10.1101/cshperspect.a025247.
3. Edoo, Z.; Arthur, M.; Hugonnet, J.E. Reversible inactivation of a peptidoglycan transpeptidase by a beta-lactam antibiotic mediated by beta-lactam-ring recyclization in the enzyme active site. *Scientific reports* **2017**, *7*, 9136, doi:10.1038/s41598-017-09341-8.
4. De Rosa, M.; Verdino, A.; Soriente, A.; Marabotti, A. The Odd Couple(s): An Overview of Beta- Lactam Antibiotics Bearing More Than One Pharmacophoric Group. *International journal of molecular sciences* **2021**, *22*, doi:10.3390/ijms22020617.
5. Strommenger, B.; Bartels, M.D.; Kurt, K.; Layer, F.; Rohde, S.M.; Boye, K.; Westh, 306 H.; Witte, W.; De Lencastre, H.; Nubel, U.

Evolution of methicillin-resistant Staphylococcus aureus towards increasing resistance. *The Journal of antimicrobial chemotherapy* **2014**, *69*, 616-622, doi:10.1093/jac/dkt413.

6. Dreier, J.; Ruggerone, P. Interaction of antibacterial compounds with RND e ffl ux pumps in Pseudomonas aeruginosa. *Frontiers in microbiology* **2015**, *6*, 660, doi:10.3389/fmicb.2015.00660.
7. Kakoullis, L.; Papachristodoulou, E.; Chra, P.; Panos, G. Mechanisms of Antibiotic Resistance in Important Gram-Positive and Gram-Negative Pathogens and Novel Antibiotic Solutions. *Antibiotics* **2021**, *10*, doi:10.3390/antibiotics10040415.
8. Lee, N.; Yuen, K.Y.; Kumana, C.R. Clinical role of beta-lactam/beta-lactamase inhibitor combinations. *Drugs* **2003**, *63*, 1511-1524, doi:10.2165/00003495-200363140-00006.
9. James, C.E.; Mahendran, K.R.; Molitor, A.; Bolla, J.M.; Bessonov, A.N.; Winterhalter, M.; Pages, J.M. How beta-lactam antibiotics enter bacteria: a dialogue with the porins. *PloS one* **2009**, *4*, e5453, doi:10.1371/journal.pone.0005453.
10. Munita, J.M.; Arias, C.A. Mechanisms of Antibiotic Resistance. *Microbiology spectrum* **2016**, *4*, doi:10.1128/microbiolspec.VMBF-0016-2015.
11. Kong, K.F.; Schneper, L.; Mathee, K. Beta-lactam antibiotics: from antibiosis to resistance and bacteriology. *APMIS : acta pathologica, microbiologica, et immunologica Scandinavica* **2010**, *118*, 1- 36, doi:10.1111/j.1600-0463.2009.02563.x.
12. Duplessis, C.; Crum-Cianflone, N.F. Ceftaroline: A New Cephalosporin with Activity against Methicillin-Resistant Staphylococcus aureus (MRSA). *Clinical medicine reviews in therapeutics* **2011**, *3*, doi:10.4137/CMRT.S1637.
13. Abbas, M.; Paul, M.; Huttner, A. New and improved? A review of novel antibiotics for Gram-positive bacteria. *Clinical microbiology and infection : the official publication of the European Society of Clinical Microbiology and Infectious Diseases* **2017**, *23*, 697-703, doi:10.1016/j.cmi.2017.06.010.
14. Papp-Wallace, K.M.; Endimiani, A.; Taracila, M.A.; Bonomo, R.A. Carbapenems: past, present, andfuture. *Antimicrobial agents and chemotherapy* **2011**, *55*, 4943-4960, doi:10.1128/AAC.00296-11.
15. Elshamy, A.A.; Aboshanab, K.M. A review on bacterial resistance to carbapenems: epidemiology, detection and treatment options. *Future science OA* **2020**, *6*, FSO438, doi:10.2144/fsoa-2019-0098.
16. Spera, A.M.; Esposito, S.; Pagliano, P. Emerging antibiotic resistance: carbapenemase-producing enterobacteria. Bad new bugs, still no new drugs. *Le infezioni in medicina* **2019**, *27*, 357-364.
17. Nordmann, P.; Dortet, L.; Poirel, L. Carbapenem resistance in Enterobacteriaceae: here is the storm! *Trends in molecular medicine* **2012**, *18*, 263-272, doi:10.1016/j.molmed.2012.03.003.
18. Pokharel, K.; Dawadi, B.R.; Bhatt, C.P.; Gupte, S.; Jha, B. Resistance Pattern of Carbapenem onEnterobacteriaceae. *JNMA; journal of the Nepal Medical Association* **2018**, *56*, 931-935.
19. Abdelaziz, S.M.; Aboshanab, K.M.; Yahia, I.S.; Yassien, M.A.; Hassouna, N.A. Correlation between the Antibiotic Resistance Genes and Susceptibility to Antibiotics among the Carbapenem-Resistant Gram-Negative Pathogens. *Antibiotics* **2021**, *10*, doi:10.3390/antibiotics10030255.
20. Alizadeh, N.; Ahangarzadeh Rezaee, M.; Samadi Kafil, H.; Hasani, A.; Soroush Barhaghi, M.H.; Milani, M.; Yeganeh Sefidan, F.; Memar, M.Y.; Lalehzadeh, A.; Ghotaslou, R. Evaluation of Resistance Mechanisms in Carbapenem-Resistant Enterobacteriaceae. *Infection and drug resistance* **2020**, *13*, 1377-1385, doi:10.2147/IDR.S244357.
21. Lasko, M.J.; Nicolau, D.P. Carbapenem-Resistant Enterobacterales: Considerations for Treatment in the Era of New Antimicrobials and Evolving Enzymology. *Current infectious disease reports* **2020**, *22*, 6, doi:10.1007/s11908-020-0716-3.
22. Codjoe, F.S.; Donkor, E.S. Carbapenem Resistance: A Review. *Medical sciences* **2017**, *6*, doi:10.3390/medsci6010001.
23. Heo, Y.A. Imipenem/Cilastatin/Relebactam: A Review in Gram-Negative Bacterial Infections. *Drugs* **2021**, *81*, 377-388, doi:10.1007/s40265-021-01471-8.
24. Xu, C.; Wang, D.; Zhang, X.; Liu, H.; Zhu, G.; Wang, T.; Cheng, Z.; Wu, W.; Bai, F.; Jin, Y. Mechanisms for Rapid Evolution of Carbapenem Resistance in a Clinical Isolate of Pseudomonas aeruginosa. *Frontiers in microbiology* **2020**, *11*, 1390, doi:10.3389/fmicb.2020.01390.
25. Indrajith, S.; Mukhopadhyay, A.K.; Chowdhury, G.; Farraj, D.A.A.; Alkufeidy, 357 R.M.; Natesan, S.; Meghanathan, V.; Gopal,

S.; Muthupandian, S. Molecular insights of Carbapenem resistance Klebsiella pneumoniae isolates with focus on multidrug resistance from clinical samples. *Journal of infection and public health* **2021**, *14*, 131-138, doi:10.1016/j.jiph.2020.09.018.
26. Zhang, Y.; Jiang, X.; Wang, Y.; Li, G.; Tian, Y.; Liu, H.; Ai, F.; Ma, Y.; Wang, B.; Ruan, F., et al. Contribution of beta-lactamases and porin proteins OmpK35 and OmpK36 to carbapenem resistance in clinical isolates of KPC-2-producing Klebsiella pneumoniae. *Antimicrobial agents and chemotherapy* **2014**, *58*, 1214-1217, doi:10.1128/AAC.02045-12.
27. Poole, K. Efflux pumps as antimicrobial resistance mechanisms. *Annals of medicine* **2007**, *39*, 162- 176, doi:10.1080/07853890701195262.
28. Fournier, D.; Richardot, C.; Muller, E.; Robert-Nicoud, M.; Llanes, C.; Plesiat, P.; Jeannot, K. Complexity of resistance mechanisms to imipenem in intensive care unit strains of Pseudomonas aeruginosa. *The Journal of antimicrobial chemotherapy* **2013**, *68*, 1772-1780, doi:10.1093/jac/dkt098.
29. Abdi, S.N.; Ghotaslou, R.; Ganbarov, K.; Mobed, A.; Tanomand, A.; Yousefi, M.; Asgharzadeh, M.; Kafil, H.S. Acinetobacter baumannii Efflux Pumps and Antibiotic Resistance. *Infection and drug resistance* **2020**, *13*, 423-434, doi:10.2147/IDR.S228089.
30. Diene, S.M.; Rolain, J.M. Carbapenemase genes and genetic platforms in Gram-negative bacilli: Enterobacteriaceae, Pseudomonas and Acinetobacter species. *Clinical microbiology and infection : e official publication of the European Society of Clinical Microbiology and Infectious Diseases* **2014**, *20*, 831-838, doi:10.1111/1469-0691.12655.
31. Reyes, J.A.; Melano, R.; Cardenas, P.A.; Trueba, G. Mobile genetic elements associated with carbapenemase genes in South American Enterobacterales. *The Brazilian journal of infectious diseases : an official publication of the Brazilian Society of Infectious Diseases* **2020**, *24*, 231-238, doi:10.1016/j.bjid.2020.03.002.
32. Sawa, T.; Kooguchi, K.; Moriyama, K. Molecular diversity of extended-spectrum beta-lactamases and carbapenemases, and antimicrobial resistance. *Journal of intensive care* **2020**, *8*, 13, doi:10.1186/s40560-020-0429-6.
33. Bush, K.; Jacoby, G.A. Updated functional classification of beta-lactamases. *Antimicrobial agents and chemotherapy* **2010**, *54*, 969-976, doi:10.1128/AAC.01009-09.
34. Tooke, C.L.; Hinchliffe, P.; Bragginton, E.C.; Colenso, C.K.; Hirvonen, V.H.A.; Takebayashi, Y.; Spencer, J. beta-Lactamases and beta-Lactamase Inhibitors in the 21st Century. *Journal of molecular biology* **2019**, *431*, 3472-3500, doi:10.1016/j.jmb.2019.04.002.
35. Qadri, H.; Haseeb, A.; Mir, M. Novel Strategies to Combat the Emerging Drug Resistance in Human Pathogenic Microbes. *Current drug targets* **2020**, 10.2174/1389450121666201228123212, doi:10.2174/1389450121666201228123212.
36. Vrancianu, C.O.; Gheorghe, I.; Dobre, E.G.; Barbu, I.C.; Cristian, R.E.; Popa, M.; Lee, S.H.; Limban, C.; Vlad, I.M.; Chifiriuc, M.C. Emerging Strategies to Combat beta-Lactamase Producing ESKAPE Pathogens. *International journal of molecular sciences* **2020**, *21*, doi:10.3390/ijms21228527.
37. Yuan, J.; Chow, D.C.; Huang, W.; Palzkill, T. Identification of a beta-lactamase inhibitory protein variant that is a potent inhibitor of Staphylococcus PC1 beta-lactamase. *Journal of molecular biology* **2011**, *406*, 730-744, doi:10.1016/j.jmb.2011.01.014.
38. Rawat, D.; Nair, D. Extended-spectrum beta-lactamases in Gram Negative Bacteria. *Journal of global infectious diseases* **2010**, *2*, 263-274, doi:10.4103/0974-777X.68531.
39. Chaves, J.; Ladona, M.G.; Segura, C.; Coira, A.; Reig, R.; Ampurdanes, C. SHV-1 beta-lactamase ismainly a chromosomally encoded species-specific enzyme in Klebsiella pneumoniae. *Antimicrobialagents and chemotherapy* **2001**,
40. Rossolini, G.M.; D'Andrea, M.M.; Mugnaioli, C. The spread of CTX-M-type extended-spectrum beta lactamases. *Clinical microbiology and infection : the official publication of the European Society of Clinical Microbiology and Infectious Diseases* **2008**, *14 Suppl 1*, 33-41, doi:10.1111/j.1469- 0691.2007.01867.x.
41. Rapp, R.P.; Urban, C. Klebsiella pneumoniae carbapenemases in Enterobacteriaceae: history, evolution, and microbiology concerns. *Pharmacotherapy* **2012**, *32*, 399-407, doi:10.1002/j.1875- 9114.2012.01035.x.
42. Coque, T.M.; Oliver, A.; Perez-Diaz, J.C.; Baquero, F.; Canton, R. Genes encoding TEM-4, SHV-2, and CTX-M-10 extended-spectrum beta-lactamases are carried by multiple Klebsiella pneumoniae clones in a single hospital (Madrid, 1989 to 2000). *Antimicrobial agents and chemotherapy* **2002**,*46*, 500-510, doi:10.1128/AAC.46.2.500-510.2002.
43. Liakopoulos, A.; Mevius, D.; Ceccarelli, D. A Review of SHV Extended-Spectrum beta-Lactamases: Neglected Yet Ubiquitous.

*Frontiers in microbiology* **2016**, *7*, 1374, doi:10.3389/fmicb.2016.01374.

44. De Angelis, G.; Del Giacomo, P.; Posteraro, B.; Sanguinetti, M.; Tumbarello, M. Molecular Mechanisms, Epidemiology, and Clinical Importance of beta-Lactam Resistance in Enterobacteriaceae. *International journal of molecular sciences* **2020**, *21*, doi:10.3390/ijms21145090.
45. Karaiskos, I.; Giamarellou, H. Carbapenem-Sparing Strategies for ESBL Producers: When and How. *Antibiotics* **2020**, *9*, doi:10.3390/antibiotics9020061.
46. Naas, T.; Dortet, L.; Iorga, B.I. Structural and Functional Aspects of Class A Carbapenemases. *Current drug targets* **2016**, *17*, 1006-1028, doi:10.2174/1389450117666160310144501.
47. Hammoudi Halat, D.; Ayoub Moubareck, C. The Current Burden of Carbapenemases: Review of Significant Properties and Dissemination among Gram-Negative Bacteria. *Antibiotics* **2020**, *9*, doi:10.3390/antibiotics9040186.
48. Swathi, C.H.; Chikala, R.; Ratnakar, K.S.; Sritharan, V. A structural, epidemiological & genetic overview of Klebsiella pneumoniae carbapenemases (KPCs). *The Indian journal of medical research* **2016**, *144*, 21-31, doi:10.4103/0971-5916.193279.
49. Mukherjee, S.; Mitra, S.; Dutta, S.; Basu, S. Neonatal Sepsis: The Impact of Carbapenem-Resistant and Hypervirulent Klebsiella pneumoniae. *Frontiers in medicine* **2021**, *8*, 634349, doi:10.3389/fmed.2021.634349.
50. Potron, A.; Poirel, L.; Nordmann, P. Emerging broad-spectrum resistance in Pseudomonas aeruginosa and Acinetobacter baumannii: Mechanisms and epidemiology. *International journal of antimicrobial agents* **2015**, *45*, 568-585, doi:10.1016/j.ijantimicag.2015.03.001.
51. Reck, F.; Bermingham, A.; Blais, J.; Capka, V.; Cariaga, T.; Casarez, A.; Colvin, R.; Dean, C.R.; Fekete, A.; Gong, W., et al. Optimization of novel monobactams with activity against carbapenem-resistant Enterobacteriaceae - Identification of LYS228. *Bioorganic & medicinal chemistry letters* **2018**, *28*, 748-755, doi:10.1016/j.bmcl.2018.01.006.
52. Yoon, E.J.; Jeong, S.H. Mobile Carbapenemase Genes in Pseudomonas aeruginosa. *Frontiers in microbiology* **2021**, *12*, 614058, doi:10.3389/fmicb.2021.614058.
53. Nordmann, P.; Naas, T.; Poirel, L. Global spread of Carbapenemase-producing Enterobacteriaceae. *Emerging infectious diseases* **2011**, *17*, 1791-1798, doi:10.3201/eid1710.110655.
54. Tan, X.; Kim, H.S.; Baugh, K.; Huang, Y.; Kadiyala, N.; Wences, M.; Singh, N.; Wenzler, E.; Bulman, Z.P. Therapeutic Options for Metallo-beta-Lactamase-Producing Enterobacterales. *Infection and drug resistance* **2021**, *14*, 125-142, doi:10.2147/IDR.S246174.
55. Poirel, L.; Hombrouck-Alet, C.; Freneaux, C.; Bernabeu, S.; Nordmann, P. Global spread of New Delhi metallo-beta-lactamase 1. *The Lancet. Infectious diseases* **2010**, *10*, 832, doi:10.1016/S1473- 3099(10)70279-6.
56. Bedenic, B.; Plecko, V.; Sardelic, S.; Uzunovic, S.; Godic Torkar, K. Carbapenemases in gram-negative bacteria: laboratory detection and clinical significance. *BioMed research international* **2014**, *2014*, 841951, doi:10.1155/2014/841951.
57. Hong, J.; Kang, D.; Kim, D. Performance Evaluation of the Newly Developed In Vitro Rapid Diagnostic Test for Detecting OXA-48-Like, KPC-, NDM-, VIM- and IMP-Type Carbapenemases: The RESIST-5 O.K.N.V.I. Multiplex Lateral Flow Assay. *Antibiotics* **2021**, *10*, doi:10.3390/antibiotics10040460.
58. Dortet, L.; Poirel, L.; Nordmann, P. Worldwide dissemination of the NDM-type carbapenemases in Gram-negative bacteria. *BioMed research international* **2014**, *2014*, 249856, doi:10.1155/2014/249856.
59. Kohler, P.; Tijet, N.; Kim, H.C.; Johnstone, J.; Edge, T.; Patel, S.N.; Seah, C.; Willey, B.; Coleman, B.; Green, K., et al. Dissemination of Verona Integron-encoded Metallo-beta-lactamase among clinical and environmental Enterobacteriaceae isolates in Ontario, Canada. *Scientific reports* **2020**, *10*, 18580, doi:10.1038/s41598-020-75247-7.
60. Hishinuma, T.; Tada, T.; Uchida, H.; Shimojima, M.; Kirikae, T. A Novel VIM-Type Metallo-beta-Lactamase Variant, VIM-60, with Increased Hydrolyzing Activity against Fourth-GenerationCephalosporins in Pseudomonas aeruginosa Clinical Isolates in Japan. *Antimicrobial agents andchemotherapy* **2019**, *63*, doi:10.1128/AAC.00124-19.
61. Heiden, S.E.; Sydow, K.; Schaefer, S.; Klempien, I.; Balau, V.; Bauer, P.; Hubner, N.O.; Schaufler, K. Nearly Identical Plasmids Encoding VIM-1 and Mercury Resistance in Enterobacteriaceae from North-Eastern Germany. *Microorganisms* **2021**, *9*, doi:10.3390/microorganisms9071345.

62. McGeary, R.P.; Tan, D.T.; Schenk, G. Progress toward inhibitors of metallo-beta-lactamases. *Future medicinal chemistry* **2017**, *9*, 673-691, doi:10.4155/fmc-2017-0007.
63. Gupta, V.; Kumarasamy, K.; Gulati, N.; Garg, R.; Krishnan, P.; Chander, J. AmpC beta-lactamases in nosocomial isolates of Klebsiella pneumoniae from India. *The Indian journal of medical research* **2012**, *136*, 237-241.
64. Jacoby, G.A. AmpC beta-lactamases. *Clinical microbiology reviews* **2009**, *22*, 161-182, Table of Contents, doi:10.1128/CMR.00036-08.
65. Herrmann, L.; Kimmig, A.; Rodel, J.; Hagel, S.; Rose, N.; Pletz, M.W.; Bahrs, C. Early Treatment Outcomes for Bloodstream Infections Caused by Potential AmpC Beta-Lactamase-Producing Enterobacterales with Focus on Piperacillin/Tazobactam: A Retrospective Cohort Study. *Antibiotics* **2021**, *10*, doi:10.3390/antibiotics10060665.
66. Tan, S.H.; Ng, T.M.; Chew, K.L.; Yong, J.; Wu, J.E.; Yap, M.Y.; Heng, S.T.; Ng, W.H.W.; Wan, S.; Cheok, S.J.H., et al. Outcomes of treating AmpC-producing Enterobacterales bacteraemia with carbapenems vs. non-carbapenems. *International journal of antimicrobial agents* **2020**, *55*, 105860, doi:10.1016/j.ijantimicag.2019.105860.
67. Song, W.; Kim, J.S.; Kim, H.S.; Yong, D.; Jeong, S.H.; Park, M.J.; Lee, K.M. Increasing trend in the prevalence of plasmid-mediated AmpC beta-lactamases in Enterobacteriaceae lacking chromosomal ampC gene at a Korean university hospital from 2002 to 2004. *Diagnostic microbiology and infectious disease* **2006**, *55*, 219-224, doi:10.1016/j.diagmicrobio.2006.01.012.
68. Rensing, K.L.; Abdallah, H.M.; Koek, A.; Elmowalid, G.A.; Vandenbroucke-Grauls, C.; Al Naiemi, N.; van Dijk, K. Prevalence of plasmid-mediated AmpC in Enterobacteriaceae isolated from humans and from retail meat in Zagazig, Egypt. *Antimicrobial resistance and infection control* **2019**, *8*, 45, doi:10.1186/s13756-019-0494-6.
69. Mata, C.; Miro, E.; Rivera, A.; Mirelis, B.; Coll, P.; Navarro, F. Prevalence of acquired AmpC beta lactamases in Enterobacteriaceae lacking inducible chromosomal ampC genes at a Spanish hospital from 1999 to 2007. *Clinical microbiology and infection : the official publication of the European Society of Clinical Microbiology and Infectious Diseases* **2010**, *16*, 472-476, doi:10.1111/j.1469-0691.2009.02864.x.
70. Hanson, N.D.; Sanders, C.C. Regulation of inducible AmpC beta-lactamase expression among Enterobacteriaceae. *Current pharmaceutical design* **1999**, *5*, 881-894.
71. D'Souza, R.; Nguyen, L.P.; Pinto, N.A.; Lee, H.; Vu, T.N.; Kim, H.; Cho, H.S.; Yong, D. Role of AmpG in the resistance to beta-lactam agents, including cephalosporins and carbapenems: candidate for a novel antimicrobial target. *Annals of clinical microbiology and antimicrobials* **2021**, *20*, 45, doi:10.1186/s12941-021-00446-7.
72. Antunes, N.T.; Fisher, J.F. Acquired Class D beta-Lactamases. *Antibiotics* **2014**, *3*, 398-434, doi:10.3390/antibiotics3030398.
73. Antunes, N.T.; Lamoureaux, T.L.; Toth, M.; Stewart, N.K.; Frase, H.; Vakulenko, S.B. Class D beta lactamases: are they all carbapenemases? *Antimicrobial agents and chemotherapy* **2014**, *58*, 2119- 2125, doi:10.1128/AAC.02522-13.
74. Vazquez-Ucha, J.C.; Arca-Suarez, J.; Bou, G.; Beceiro, A. New Carbapenemase Inhibitors: Clearing the Way for the beta-Lactams. *International journal of molecular sciences* **2020**, *21*, doi:10.3390/ijms21239308.
75. Livermore, D.M.; Day, M.; Cleary, P.; Hopkins, K.L.; Toleman, M.A.; Wareham, D.W.; Wiuff, C.; Doumith, M.; Woodford, N. OXA-1 beta-lactamase and non-susceptibility to penicillin/beta lactamase inhibitor combinations among ESBL-producing Escherichia coli. *The Journal of antimicrobial chemotherapy* **2019**, *74*, 326-333, doi:10.1093/jac/dky453.
76. Rivera-Izquierdo, M.; Lainez-Ramos-Bossini, A.J.; Rivera-Izquierdo, C.; Lopez-Gomez, J.; Fernandez- Martinez, N.F.; Redruello-Guerrero, P.; Martin-delosReyes, L.M.; Martinez-Ruiz, V.; Moreno-Roldan, E.; Jimenez-Mejias, E. OXA-48 Carbapenemase-Producing Enterobacterales in Spanish Hospitals: An Updated Comprehensive Review on a Rising Antimicrobial Resistance. *Antibiotics* **2021**, *10*, doi:10.3390/antibiotics10010089.
77. Stewart, A.; Harris, P.; Henderson, A.; Paterson, D. Treatment of Infections by OXA-48-Producing Enterobacteriaceae. *Antimicrobial agents and chemotherapy* **2018**, *62*, doi:10.1128/AAC.01195-18.
78. Mairi, A.; Pantel, A.; Sotto, A.; Lavigne, J.P.; Touati, A. OXA-48-like carbapenemases producing Enterobacteriaceae in different niches. *European journal of clinical microbiology & infectious diseases : official publication of the European Society of Clinical Microbiology* **2018**, *37*, 587-604, doi:10.1007/s10096-017-3112-7.

79. Mentasti, M.; Prime, K.; Sands, K.; Khan, S.; Wootton, M. Rapid detection of OXA-23-like, OXA-24- like, and OXA-58-like carbapenemases from Acinetobacter species by real-time PCR. *The Journal of hospital infection* **2020**, *105*, 741-746, doi:10.1016/j.jhin.2020.06.015.
80. Poirel, L.; Castanheira, M.; Carrer, A.; Rodriguez, C.P.; Jones, R.N.; Smayevsky, J.; Nordmann, P. OXA-163, an OXA-48-related class D beta-lactamase with extended activity toward expanded spectrum cephalosporins. *Antimicrobial agents and chemotherapy* **2011**, *55*, 2546-2551, doi:10.1128/AAC.00022-11.
81. Potron, A.; Rondinaud, E.; Poirel, L.; Belmonte, O.; Boyer, S.; Camiade, S.; Nordmann, P. Genetic and biochemical characterisation of OXA-232, a carbapenem-hydrolysing class D beta-lactamase from Enterobacteriaceae. *International journal of antimicrobial agents* **2013**, *41*, 325-329, doi:10.1016/j.ijantimicag.2012.11.007.
82. El-Housseiny, G.S.; Ibrahim, A.A.; Yassien, M.A.; Aboshanab, K.M. Production and statistical optimization of Paromomycin by Streptomyces rimosus NRRL 2455 in solid state fermentation. *BMC microbiology* **2021**, *21*, 34, doi:10.1186/s12866-021-02093-6.
83. Breijyeh, Z.; Jubeh, B.; Karaman, R. Resistance of Gram-Negative Bacteria to Current Antibacterial Agents and Approaches to Resolve It. *Molecules* **2020**, *25*, doi:10.3390/molecules25061340.
84. Khan, F.; Pham, D.T.N.; Kim, Y.M. Alternative strategies for the application of aminoglycoside antibiotics against the biofilm-forming human pathogenic bacteria. *Applied microbiology and biotechnology* **2020**, *104*, 1955-1976, doi:10.1007/s00253-020-10360-1.
85. Kotra, L.P.; Haddad, J.; Mobashery, S. Aminoglycosides: perspectives on mechanisms of action and resistance and strategies to counter resistance. *Antimicrobial agents and chemotherapy* **2000**, *44*, 3249-3256, doi:10.1128/AAC.44.12.3249-3256.2000.
86. Garneau-Tsodikova, S.; Labby, K.J. Mechanisms of Resistance to Aminoglycoside Antibiotics: Overview and Perspectives. *MedChemComm* **2016**, *7*, 11-27, doi:10.1039/C5MD00344J.
87. Skold, O. Sulfonamide resistance: mechanisms and trends. *Drug resistance updates : reviews and commentaries in antimicrobial and anticancer chemotherapy* **2000**, *3*, 155-160, doi:10.1054/drup.2000.0146.
88. Azzam, R.A.; Elsayed, R.E.; Elgemeie, G.H. Design, Synthesis, and Antimicrobial Evaluation of a New Series of N-Sulfonamide 2-Pyridones as Dual Inhibitors of DHPS and DHFR Enzymes. *ACS omega* **2020**, *5*, 10401-10414, doi:10.1021/acsomega.0c00280.
89. Griffith, E.C.; Wallace, M.J.; Wu, Y.; Kumar, G.; Gajewski, S.; Jackson, P.; Phelps, G.A.; Zheng, Z.; Rock, C.O.; Lee, R.E., et al. The Structural and Functional Basis for Recurring Sulfa Drug Resistance Mutations in Staphylococcus aureus Dihydropteroate Synthase. *Frontiers in microbiology* **2018**, *9*, 1369, doi:10.3389/fmicb.2018.01369.
90. Vilcheze, C.; Jacobs, W.R., Jr. The combination of sulfamethoxazole, trimethoprim, and isoniazid or rifampin is bactericidal and prevents the emergence of drug resistance in Mycobacterium tuberculosis. *Antimicrobial agents and chemotherapy* **2012**, *56*, 5142-5148, doi:10.1128/AAC.00832-12.
91. Falagas, M.E.; Vardakas, K.Z.; Roussos, N.S. Trimethoprim/sulfamethoxazole for Acinetobacter spp.: A review of current microbiological and clinical evidence. *International journal of antimicrobial agents* **2015**, *46*, 231-241, doi:10.1016/j.ijantimicag.2015.04.002.
92. Fair, R.J.; Tor, Y. Antibiotics and bacterial resistance in the 21st century. *Perspectives in medicinal chemistry* **2014**, *6*, 25-64, doi:10.4137/PMC.S14459.
93. Kim, D.W.; Thawng, C.N.; Lee, K.; Wellington, E.M.H.; Cha, C.J. A novel sulfonamide resistance mechanism by two-component flavin-dependent monooxygenase system in sulfonamide-degrading actinobacteria. *Environment international* **2019**, *127*, 206-215, doi:10.1016/j.envint.2019.03.046.
94. Pham, T.D.M.; Ziora, Z.M.; Blaskovich, M.A.T. Quinolone antibiotics. *MedChemComm* **2019**, *10*, 1719-1739, doi:10.1039/c9md00120d.
95. Aldred, K.J.; Kerns, R.J.; Osheroff, N. Mechanism of quinolone action and resistance. *Biochemistry* **2014**, *53*, 1565-1574, doi:10.1021/bi5000564.
96. Hooper, D.C. Mechanisms of action of antimicrobials: focus on fluoroquinolones. *Clinical infectious diseases : an official publication of the Infectious Diseases Society of America* **2001**, *32 Suppl 1*, S9- S15, doi:10.1086/319370.

97. Kapoor, G.; Saigal, S.; Elongavan, A. Action and resistance mechanisms of antibiotics: A guide for clinicians. *Journal of anaesthesiology, clinical pharmacology* **2017**, *33*, 300-305, doi:10.4103/joacp.JOACP_349_15.
98. Robicsek, A.; Jacoby, G.A.; Hooper, D.C. The worldwide emergence of plasmid-mediated quinolone resistance. *The Lancet. Infectious diseases* **2006**, *6*, 629-640, doi:10.1016/S1473-3099(06)70599-0.
99. Zhao, L.; Wang, S.; Li, X.; He, X.; Jian, L. Development of in vitro resistance to fluoroquinolones in Pseudomonas aeruginosa. *Antimicrobial resistance and infection control* **2020**, *9*, 124, doi:10.1186/s13756-020-00793-8.
100. Alekshun, M.N.; Levy, S.B. Molecular mechanisms of antibacterial multidrug resistance. *Cell* **2007**, *128*, 1037-1050, doi:10.1016/j.cell.2007.03.004.
101. Neame, M.; King, C.; Riordan, A.; Iyer, A.; Kneen, R.; Sinha, I.; Hawcutt, D.B. Seizures and quinolone antibiotics in children: a systematic review of adverse events. *European journal of hospital pharmacy : science and practice* **2020**, *27*, 60-64, doi:10.1136/ejhpharm-2018-001805.
102. Chopra, I.; Roberts, M. Tetracycline antibiotics: mode of action, applications, molecular biology, and epidemiology of bacterial resistance. *Microbiology and molecular biology reviews: MMBR* **2001**, *65*, 232-260 ; second page, table of contents, doi:10.1128/MMBR.65.2.232-260.2001.
103. Shang, Z.; Salim, A.A.; Khalil, Z.; Bernhardt, P.V.; Capon, R.J. Fungal Biotransformation of Tetracycline Antibiotics. *The Journal of organic chemistry* **2016**, *81*, 6186-6194, doi:10.1021/acs.joc.6b01272.
104. Nelson, M.L.; Levy, S.B. The history of the tetracyclines. *Annals of the New York Academy of Sciences* **2011**, *1241*, 17-32, doi:10.1111/j.1749-6632.2011.06354.x.
105. Roberts, M.C. Tetracycline therapy: update. *Clinical infectious diseases : an official publication of the Infectious Diseases Society of America* **2003**, *36*, 462-467, doi:10.1086/367622.
106. Luo, L.; Zhang, C.; Zhang, Z.; Peng, J.; Han, Y.; Wang, P.; Kong, X.; Rizwan, H.M.; Zhang, D.; Su, P., et al. Differences in Tetracycline Antibiotic Resistance Genes and Microbial Community Structure During Aerobic Composting and Anaerobic Digestion. *Frontiers in microbiology* **2020**, *11*, 583995, doi:10.3389/fmicb.2020.583995.
107. Jahantigh, M.; Samadi, K.; Dizaji, R.E.; Salari, S. Antimicrobial resistance 609 and prevalence of tetracycline resistance genes in Escherichia coli isolated from lesions of colibacillosis in broiler chickens in Sistan, Iran. *BMC veterinary research* **2020**, *16*, 267, doi:10.1186/s12917-020-02488-z.
